# Barriers and Opportunities for the Use of Digital Tools in Medicines Optimization Across the Interfaces of Care: Stakeholder Interviews in the United Kingdom

**DOI:** 10.2196/42458

**Published:** 2023-03-10

**Authors:** Clare Tolley, Helen Seymour, Neil Watson, Hamde Nazar, Jude Heed, Dave Belshaw

**Affiliations:** 1 School of Pharmacy Newcastle University Newcastle upon Tyne United Kingdom; 2 Pharmacy Department The Newcastle upon Tyne Hospitals NHS Foundation Trust Newcastle upon Tyne United Kingdom; 3 North East and North Cumbria Academic Health Science Network Newcastle upon Tyne United Kingdom

**Keywords:** health information exchange, patient safety, medicines optimization, transfer of care, health informatics, qualitative

## Abstract

**Background:**

People with long-term conditions frequently transition between care settings that require information about a patient’s medicines to be transferred or translated between systems. This process is currently error prone and associated with unintentional changes to medications and miscommunication, which can lead to serious patient consequences. One study estimated that approximately 250,000 serious medication errors occur in England when a patient transitions from hospital to home. Digital tools can equip health care professionals with the right information at the right time and place to support practice.

**Objective:**

This study aimed to answer the following questions: what systems are being used to transfer information across interfaces of care within a region of England? and what are the challenges and potential opportunities for more effective cross-sector working to support medicines optimization?

**Methods:**

A team of researchers at Newcastle University conducted a qualitative study by performing in-depth semistructured interviews with 23 key stakeholders in medicines optimization and IT between January and March 2022. The interviews lasted for approximately 1 hour. The interviews and field notes were transcribed and analyzed using the framework approach. The themes were discussed, refined, and applied systematically to the data set. Member checking was also performed.

**Results:**

This study revealed themes and subthemes pertaining to 3 key areas: transfer of care issues, challenges of digital tools, and future hopes and opportunities. We identified a major complexity in terms of the number of different medicine management systems used throughout the region. There were also important challenges owing to incomplete patient records. We also highlighted the barriers related to using multiple systems and their subsequent impact on user workflow, a lack of interoperability between systems, gaps in the availability of digital data, and poor IT and change management. Finally, participants described their hopes and opportunities for the future provision of medicines optimization services, and there was a clear need for a patient-centered consolidated integrated health record for use by all health and care professionals across different sectors, bridging those working in primary, secondary, and social care.

**Conclusions:**

The effectiveness and utility of shared records depend on the data within; therefore, health care and digital leaders must support and strongly encourage the adoption of established and approved digital information standards. Specific priorities regarding understanding of the vision for pharmacy services and supporting this with appropriate funding arrangements and strategic planning of the workforce were also described. In addition, the following were identified as key enablers to harness the benefits of digital tools to support future medicines optimization: development of minimal system requirements; enhanced IT system management to reduce unnecessary repetition; and importantly, meaningful and continued collaboration with clinical and IT stakeholders to optimize systems and share good practices across care sectors.

## Introduction

### Background

Medicines are the most common therapeutic intervention in the United Kingdom’s National Health Service (NHS). However, it is estimated that between 30% and 50% of medicines prescribed for long-term conditions are not taken as intended [1]. More than a quarter of the adult population in England live with ≥2 conditions [2], and approximately 15% of people in England take ≥5 medicines a day, with 7% taking ≥8 medicines per day [3]. Furthermore, the prevalence of multimorbidity is estimated to increase substantially, with the proportion of those with >4 diseases almost doubling to 17% by 2035 [4]. Therefore, it is important that individuals receive the maximum benefit from their medicines while minimizing harm. Effective medicines optimization contributes to improved health outcomes, patient care, safety and satisfaction, improved efficiency and use of resources, better use of professional skills, and effective clinical governance [5,6].

### Transfer of Care

People with long-term health conditions frequently transition between care settings; consequently, information about a patient’s medicines is regularly transferred or translated between systems. However, the point at which patients transfer across different interfaces of care is high risk and is associated with unintentional changes to medications, errors, and miscommunication [7]. This can have consequences for patients, health care professionals, and the health system as a whole [8]. For example, each interaction with a health care professional may result in medication or treatment changes. Problematic polypharmacy may then occur, whereby multiple medicines are prescribed inappropriately or in which the intended benefits of the prescribed medications are not realized [3].

The Department of Health and Social Care’s report, *Good for you, good for us, good for everybod*y, highlighted that to reduce overprescribing, there is a need for better shared decision-making with patients; better guidance and support for clinicians; more alternatives to medicines, such as physical and social activities and talking therapies; and more structured medication reviews for those with long-term health conditions [3]. However, these goals and initiatives must be supported by effective digital systems that are interoperable and must equip health care professionals with the right information to optimize a patient’s medications [9]. A systematic review of 13 publications found that the use of IT applications such as electronic health records (EHRs), electronic decision support tools, and electronic communication applications had a positive impact on financial and health outcomes [10]. However, contemporaneous and accurate information is often not available, with substantial local variation in practice, which can result in increased workload, duplication of tasks, and errors [11,12]. Currently, there is a complex network of different systems that contain patient health record data in distinct silos throughout a patient’s journey. There is also a range of services available to support medicines optimization activities in the United Kingdom, including those that target transitions in care. For example, the Discharge Medicines Service, New Medicines Service, and NHS Community Pharmacist Consultation Service [6,13,14]. Plans also exist to roll out the electronic prescription service to secondary care and other care settings and to develop a patient-centered consolidated medication record that can be used by health care professionals working in different settings [15]. Such records may be associated with a range of benefits including improved safety, greater flexibility, enhanced ability to respond to patient queries, reduced duplication, and lower costs [16-18]. However, their use is still at an early stage, and challenges have been identified in some studies, including problems with system reliability, technical issues, and patient concerns regarding inaccuracies and the governance around sharing data [17,19]. The government recognizes the need for information to be collected once and then shared among providers to meet an individual’s needs. Interoperability is defined as *“*the ability of two or more systems or components to exchange information and to use the information that has been exchanged*”* [20]. Nationally, a range of work is underway to enhance interoperability within health and social care settings and is a clear priority for the United Kingdom [21]. A recent policy paper, *Data saves lives: reshaping health and social care with data,* highlights commitments to introducing clear and open standards to make it easier to share data safely and efficiently across care settings [22].

### Objectives

When considering how services might be delivered in the future and the necessary digital transformation, it is important to understand the current landscape of systems including their benefits, challenges, and opportunities. In this study, we aimed to address the following questions: *what systems are being used to transfer information across interfaces of care? and what are the challenges and potential opportunities for more effective cross-sector working to support medicines optimization?*

## Methods

### Overview

The aim of this study was to engage with key stakeholders in medicines optimization and IT across the North East and North Cumbria (NENC) integrated care system (ICS) to scope out current systems related to the transfer of information across interfaces of care. ICSs were established across regions of England on July 1, 2022, and have been described as *partnerships of organizations that come together to plan and deliver joined up health and care services.* Digital solutions will be central to supporting the function and role of ICSs. In addition, we sought to identify the challenges and potential opportunities for a more effective cross-sector working to support medicines optimization and inform future priorities.

A qualitative methodology was selected to ensure gathering of a detailed understanding of participants’ experiences and perspectives. A constructivist and interpretivist approach was taken [23] together with the framework approach, which is a method developed for use in applied policy research in which there is a need to address a clear set of aims and objectives, while following an inductive approach that allows theories to develop “bottom-up” [24].

Key stakeholders in medicines optimization and digital health were invited to participate in a semistructured interview to gather their perspectives on the current medicines optimization services provided and the digital tools used to support these activities. In addition, stakeholders were asked to provide their opinions on the barriers and potential opportunities for more effective medicines optimization across sectors.

### Eligibility Criteria

We included all clinicians, managers, commissioners, and stakeholders who were or had been involved in commissioning, developing, and delivering medicines optimization–related activities and who had expertise in IT, clinical informatics, and digital health solutions used within the NHS.

### Recruitment

We used a snowball sampling approach to identify suitable key participants [25]. In the first instance, the researcher was introduced to an initial set of contacts by the senior medicines optimization pharmacist based at the NENC Academic Health Science Network and the digital transformation director for the NENC Academic Health Science Network. The researcher emailed potential participants and invited them to participate in a semistructured interview. This email also included attachments to a participant information leaflet and a consent form. The participants were required to provide consent via a web-based consent form before participating in the study. We proactively aimed to engage with individuals from a range of professional backgrounds and levels of experience to ensure that the data gathered were rich and representative. Data were collected until thematic saturation was reached, and we used an inductive approach to look for the nonemergence of new themes as interviews and analysis were conducted [25,26].

### Data Collection

Semistructured interviews were conducted by 1 researcher (CT) between January and March 2022 to explore stakeholders’ perspectives on the current medicines optimization services provided in the region and the digital tools used to support these activities, including details regarding the flow of health information exchange and interoperability reached. In addition, stakeholders were asked to provide their opinions on the barriers and potential opportunities for more effective medicines optimization across sectors. A flexible topic guide was developed that incorporated open-ended questions and prompts. The guide was shared with a team of researchers and clinicians to review and refine the data before use, and the guide was developed iteratively throughout data collection [27]. Interviews lasted for approximately 1 hour and were conducted via video call by a researcher with clinical and postgraduate level of qualitative data collection experience, at a mutually convenient time for each participant. All interviews were recorded, transcribed verbatim together with accompanying field notes, and anonymized.

### Analysis (Interviews)

Qualitative data collection and analysis were iterative, allowing themes to be generated, interpreted, explored, and disconfirming evidence identified [28]. Different data sources, for example, interviews with a range of participants, facilitated triangulation to identify where and how different data converged and diverged. The main themes and subthemes were identified using a constant comparative analysis [28]. For this purpose, data were constantly compared among interviewees to explore similarities and differences between groups and to uncover explanations for why these differences existed. Field notes contributed to the analysis by providing valuable context, for example, in which the participants used their voice to stress points or in which humor was used. Field notes were also used by the interviewer to note their own reflections and consider questions for future exploration [29]. The framework approach was used, which is a 5-staged approach to thematic analysis, enabling previous theories and insights identified through literature review or experience, to inform the development of the thematic framework, while allowing theme generation based on the data and was, therefore, open to discovering unexpected concepts based on the participants’ experiences. This was used as a complementary method along with the constant comparative analysis. Themes were discussed among team members and continually refined and applied systematically to the whole data set using the computerized software N-Vivo (QSR International). All data were analyzed by qualified members of the research staff. “Member checking” was also performed, whereby a draft of the key findings was shared with all participants, who were given a minimum of 2 weeks to provide feedback on the interpretations made and contribute any additional insight [26].

### Ethics Approval

This study was approved by the Research, Policy, Intelligence, and Ethics team at Newcastle University (reference: 17851-2021).

## Results

### Overview

A total of 22 interviews were conducted with 23 participants lasting between 38 and 75 minutes (Table 1). Two participants (a community pharmacist and general practitioner [GP]) were unable to take part in an interview, owing to clinical commitments and availability. “Member checking” resulted in 1 correction to the results (clarification regarding work underway to create a patient medication records) and provided further information regarding national initiatives currently underway to support development of shared care records, which was incorporated into the discussion and recommendations in the *Addressing Digital Gaps* section.

This study revealed a range of different systems that are used across a region to support medicines optimization activities. From the interviews, a range of themes and subthemes pertaining to three key areas were identified: (1) transfer of care issues, (2) challenges of digital tools, and (3) future hopes and opportunities.

**Table 1:** Table of participants (N=23).

Profession and Sector		Participants, n (%)
**Pharmacists (n=21)**		
	Community	4 (17)
	Hospital	5 (22)
	Primary care (GP^a^ practice, primary care network, and CCG^b^)	5 (22)
	North East Ambulance Service	2^c^ (9)
	NHS^d^ England and NHS Improvement	5 (22)
GPs		2 (9)

^a^GP: general practitioner.

^b^CCG: clinical commissioning group.

^c^One interview conducted with both participants.

^d^NHS: National Health Service.

### Medicines Optimization Systems

Informed by participant interviews and relevant literature, a simplified overview of the key systems related to medicines across primary, secondary, tertiary, and social care across the NENC was developed (Multimedia Appendix 1 [30-32]). This was not intended to provide a comprehensive overview of all systems used throughout the region but instead to illustrate the complexity of how data are stored and moved between settings. Several patient health record systems have been used in general practice and primary care. A range of different community pharmacy patient medication record systems was also identified, which lacked the ability to directly transfer information with GP systems. At the time of data collection, some hospital trusts in the region used paper-based health records and prescriptions. The transfer of information between different systems and care settings was largely facilitated through bespoke solutions delivered by third-party companies in response to a particular problem, for example, a digital referral as part of the Discharge Medicines Service from 1 hospital to a community pharmacy was typically either sent using NHS mail or a website or integrated web platform such as PharmOutcomes or Cegedim. The information flow between the systems was found to be typically unidirectional.

### Transfer of Care Issues

All participants highlighted problems owing to incomplete patient records. Data were described as being held in separate silos by the GP, secondary care providers, or a community pharmacy, with ineffective data flow among them, resulting in reduced efficiency and safety. For example, participants described discrepancies between the allergy status recorded in different clinical systems (eg, missing documented allergies) and omissions owing to poor communication and clinical handover. Incomplete records also made it difficult to proactively provide care by identifying patterns of behavior that would warrant further investigation or management, for example, “if someone’s getting emergency contraception on a regular basis [from a community pharmacy], actually that should be flagging up a risk” (GP 006). A GP recalled a “significant incident where the GP hadn’t put down [methotrexate] or- on the GP record was not methotrexate, and they [the patient] ended up in an ITU in Wales and [the staff] didn’t know that the patient was on methotrexate and had actually accidentally overdosed*”* (GP 006), but the staff were unaware that the patient was even prescribed this medication*.*

### Challenges of Digital Tools

Participants raised concerns on specific challenges associated with the digital tools used to optimize medications. These included the use of multiple systems and workflow, interoperability, digital gaps, IT systems management, and change management.

#### Multiple Systems and Workflow

Participants working across all settings described the need to interact with multiple IT systems as a part of their day-to-day role. Those working in general practice, for example, would access a core EHR system alongside other systems for viewing additional information, such as hospital notes, appointment letters, or blood tests. Community pharmacists described navigating between a growing number of different systems to fulfill different tasks and purposes (refer to the quote below) and felt that exposure to “more interfaces, [presented] [...] more opportunities [...] for information to have to be re-transcribed [and] [...] actually alert tasks to get dropped, because the right people can’t see [what needs to be done]” (Pharmacist 010). They emphasized how *“*the more you’re having to step out of your day-to-day workflows and go, ‘Oh my goodness, I really must remember to send a DMS [Discharge Medicines Service] referral for this thing’. You’re just not going to do it” (Pharmacist 010):

On a Sunday, I do a COVID clinic, so I've got Q-Flow open, which is the appointments booking system [...] I’ve got PharmOutcomes open for CPCS referrals via 111. I've got PharmOutcomes open for other things, any other bits and pieces that might come. I've got Outcomes4Health, which is the sister platform. That’s open for recording the COVID vaccinations. Then we've got the...What else is there? There are half a dozen different things there. Of course, in the pharmacy clinical system, we [they] use Positive Solutions Analyst, but we’d have that open as well.

Pharmacist 007

#### Interoperability and Safety

A GP described how community nurse practitioners do not have access to the full EHR of certain patients in their care. This occurred because the community nurses were employed by the local hospital rather than by the GP practice, and the 2 organizations used different IT systems, which did not share data in real time. Consequently, automated checks such as drug-laboratory checks or drug-interaction checks are not reliable. There was the risk that if community nurses “don’t have the blood results in their system [so] they will not get that [computerised] warning, so they may merrily go ahead and prescribe that [medication]. Then conversely, if they’ve prescribed a drug, I [the GP] don’t know that the interactions are there now” (GP 012).

#### Digital Gaps

The participants revealed important gaps in the availability of health data that could not be easily shared or used by health care professionals, for example, paper-based hospital notes. A locally shared health record for people living in NENC known as the “Great North Care Record” (GNCR) is currently in development, with the aim of providing health and care workers access to current medical information. However, 1 GP highlighted how their local acute hospital trust only imported some “very, very, primitive data” into the GNCR and so was not “hugely valuable” (GP 012). In addition, data from social care, for example, care homes, were not imported into the GNCR, which meant that “any changes that are made there are not displayed” (GP 006). In contrast, however, more digitally advanced hospitals transferred a greater quantity of information from their EHR into the GNCR, which was “more useful” (GP 012). This raised the possibility of inequalities among health care providers because organizations “that are [digitally] further behind are the ones that...The Great North Care Record doesn’t help” (GP 012).

Some professional sectors, such as community pharmacy, were also unable to access the GNCR, which made it “really difficult” to maximize use of that workforce and “shift patients away from some of those higher acuity services” (Pharmacist 003). Furthermore, information governance arrangements for accessing multiple systems were considered problematic. For example, 1 pharmacist suggested that the current consent model whereby community pharmacists must obtain consent before accessing information within the summary care record was inappropriate because “if you’ve got a patient’s prescription it should almost imply by informed consent, they’ve given you their script” (Pharmacist 005).

Participants also revealed a low adoption of standardized digital codes for documenting clinical data within health systems. There are “a lot of trusts [that]don’t use dm+d” (Pharmacist 002), and many hospitals are “not using SNOMED at the moment” (GP 006); without adoption of the standards, large initiatives such as shared patient medication records are “not going to work” (Pharmacist 002). Dictionary of medicines and devices is a dictionary of descriptions and codes, which represent medicines and devices used across the NHS. Systematized Nomenclature of Medicine Clinical Terms is a structured clinical vocabulary for use in EHRs and covers diagnoses, procedures, etc.

Finally, the participants noted how certain services were being rolled out without a supportive digital infrastructure. For example, the national hypertension case finding service in community pharmacies lacked “a national system for reporting it [blood pressure] [and reporting] between the two [pharmacy to GP]” (Pharmacist 011). Consequently, a range of different communication techniques, including email, letter, or pilot digital platforms, were being used, depending on local arrangements, and as data were not collected in a standardized digital format, it could not be transferred between care settings in an interoperable way.

#### IT Systems Management

Participants suggested that a lack of minimum standards or mandating how services are technically delivered is problematic and contributes to low adoption and delays in rolling out clinical services and digital solutions:

So pharmacy DMS transmissions, we launched the DMS service but pharmacies [are] not seeing many of them because yes you can do it by snail mail and NHS Mail, but that’s not good enough. We shouldn’t launch a service without a platform to deliver it on. So every hospital should be told, “You can have your own, but we’re launching this new service and you must be able to provide a digital solution that’s integrated.”

Pharmacist 005

A need was identified to rationalize the number of digital systems used, create clear expectations for suppliers, and develop standards and frameworks that outline how services should be digitally enabled. This would reduce waste from “reinventing the wheel every single time*”* (Pharmacist 020). A new service or digital tool was developed to limit duplication and unnecessary costs. Another pharmacist added the following:

I think there needs to be a suite of expectations that everybody needs to have and it’s the same with hospitals, you can’t have a hospital system that doesn’t do these 10 things [...] every system needs to do that by a certain deadline.

Pharmacist 005

#### Change Management

Change management was seen as important for managing the future development of digital medicines optimization services. For instance, although the clinical terminology standards have now been defined in the Systematized Nomenclature of Medicine (SNOMED) and dictionary of medicines and devices (dm+d), “the hard bit is to come, which is the adoption and more importantly the transformation around that. There’s also a Hearts and Minds piece” (Pharmacist 002). Similarly, participants described concerns regarding data sharing, for instance:

If you suggest [data sharing] to some general practices, they’ll say: “No chance. Nobody should have access to that data.” But once you get over that barrier, and there's a bit of trust built into it, then you can start adding to it.

Pharmacist 003

Instead, all health care professionals need to work together to “best serve the needs of the patients” (Pharmacist 019).

### Future Hopes and Opportunities

#### Consolidated Integrated Care Record

Participants revealed several ambitions for the future of digital medicines optimization services, although the need for a single shared consolidated medication record, giving all health and care professionals access to data, was a clear priority and reflected those working across care sectors:

One record across all organizations, that’s the blue-sky thinking. The data would sit in a data repository that would be coded and accessible, via APIs, via front-end systems that could be customised to be targeted to how GPs work, targeted too how secondary care clinicians work, targeted for acute and outpatient mental health. Fundamentally, all the data would be held in one central repository for that patient and all of the systems can pull in all that data. A patient, for example from a prescribing basis, would have one prescribing record. That prescribing record would continue out of hospital, into hospital.

GP 012

This would *“*open up a number of opportunities [...] for optimisation and proper management of patients and overprescribing” (Pharmacist 002). Furthermore, by identifying and using common digital architectures and standards, such records may be linked in the future.

Some participants were worried that increased access to information could be problematic in some situations, for example, community pharmacists could have “too much information, to make a decision on” because “you could spend hours and hours and hours trailing back through communications and stuff that gets put onto records, [which] it’s probably completely inappropriate to the query” (Pharmacist 018). Consequently, several participants suggested involving end users in the development of shared records and posed that for “a sector and a workforce like community pharmacy, I think it’s big enough to warrant having a bespoke solution developed for it” (Pharmacist 001). A change in funding arrangements was also discussed as vital to support and incentivize community pharmacists to deliver clinical services at scale and to justify the need for access to shared care records in the first instance.

#### Uses of Data

There were hopes that the enhanced availability of medication data could improve workflows across care settings and population health management. Improved efficiency may be realized, for example, by sending a coded list of a patient’s medications from one system to another, so that a clinician only has to “go click, click, click, and it populates the prescribing system” (Pharmacist 010). In addition, to support better interprofessional working and better continuity of care, participants thought it would be good if they could send a “request, where you’ve got specific things that you want following up” (Pharmacist 010) alongside any information and context about a patient’s medication, directly between systems.

Participants also discussed how the development of a comprehensive shared patient record could serve as a “population health platform” (Pharmacist 002). It would then be possible to “start interrogating the information at a patient level, but [also] at a population level” (Pharmacist 002) and target public health challenges such as “overprescribing, opiate prescribing, valproate [prescribing in pregnancy]” (Pharmacist 002). Digital tools could also support better clinical prioritization, for example:

If you've got 100 Primary Care Network pharmacists delivering a structured medication review every year or so, that's 1,000 reviews a week. How do you know which 1,000 patients put in for those slots? How do you caseload? That's really important.

Pharmacist 016

Comprehensive patient data repositories could enable clinicians to develop robust strategies to identify patients more efficiently, to avoid “each practice pharmacist going out and trying to design their own searches” (Pharmacist 013). It was clear that there are major opportunities arising from more effective data sharing, and as 1 pharmacist remarked, there are likely innovations that health care professionals have not even started to dream about.

## Discussion

### Principal Findings

This research has identified several challenges and potential opportunities related to the use of digital tools for delivering medicines optimization, which are categorized under 3 key concepts: transfer of care issues, challenges of digital tools, and future hopes and opportunities. There is substantial complexity in the number of various medicines management systems used throughout the region and the challenges associated with incomplete patient records. The use of multiple systems also affects the user workflow. There was a lack of intrasystem and intersystem interoperability and important gaps in digital data in some settings (eg, social care and in some areas where hospital prescribing was paper based). Several problems related to IT systems management and change management have also been described. Participants described a clear need for a patient-centered consolidated integrated health record for use by all health and care professionals across different sectors, bridging those working in primary, secondary, and social care.

We also identified a series of recommendations relevant to health service managers, policy makers, and clinical staff, which are discussed in Table 2.

**Table 2:** Summary table: stakeholder recommendations and objectives.

Recommendations and specific objectives		Key stakeholder group
**Identify the future vision for pharmacy services and support with appropriate funding plan**		
	Support realization of the vision through appropriate strategic planning and funding arrangements, for example, community pharmacy contractual framework	Policy makers and service managers
**Development of a patient-centered consolidated integrated health record for use by health and care professionals**		
	Support digitization of social care, for example, implementation of electronic prescribing and medication administration in care homes	Researchers and service managers
	Creation of local multistakeholder working groups to ensure cocreation of digital solutions and facilitate effective teamwork and “buy-in”	Service managers
	In-depth exploration of concerns around data sharing and governance considerations with key clinical and IT stakeholders and patients and members of the public	Policy makers, service managers, researchers, clinical staff, and public
	Explore need for tailored views for health professional groups to shared care records	Service managers and researchers
	Support adoption of digital solutions across NENC^a^ region’s hospitals with a pledge to end paper records and prescribing	Policy makers, service managers, and clinical staff
**Adoption of medication standards across NHS^b^ (ie, dm+d^c^ and SNOMED CT^d^)**		
	Identify what is the level of dm+d adoption across the region	Policy makers and service managers (this is in progress via NHS Digital)
	Explore the local facilitators and barriers to adoption of standards	Policy makers, service managers, and researchers
	Prioritize communication around the need to adopt standards locally	Policy makers, service managers, and clinical staff
	Support sites through the transformation process, using tools from NHS England or FCI^e^	Service managers
**Rationalizing number of systems and services to reduce unnecessary repetition**		
	Mapping process to identify services provided across sectors and highlight duplication and gaps	Service managers and clinical staff
	Identify clinical services that would benefit from digitization in the community pharmacy sector and to support service provision and service management or audit	Service managers and researchers
**Development of frameworks and minimum standards outlining how services should be delivered using digital means at a local and national level**		
	Monitor guidance and toolkits from NHS organizations (eg, NHS Transformation Directorate) and publish updates, publish bulletins, and organize workshops to increase awareness of an organization’s responsibilities and share lessons across the region	Policy makers, service managers, researchers, and clinical staff
	Use and promote the use of forums for communication across all sectors and levels from manager to frontline staff	Service managers and clinical staff
**Cross-sector communication around approaches to digital medicines optimization**		
	Development or use of established forums and groups to proactively collate and communicate examples of good practices between different clinical and IT stakeholders	Policy makers, service managers, and researchers
**Working with suppliers to develop integrated solutions to avoid unnecessary development of bespoke solutions**		
	Share lessons across organizations about successful collaborations with suppliers on innovation projects	Service managers, researchers, and clinical staff
	Harness insight from existing projects to support scale-up of innovations	Service managers and researchers

^a^NENC: North East and North Cumbria.

^b^NHS: National Health Service.

^c^dm+d: dictionary of medicines and devices.

^d^SNOMED CT: Systematized Nomenclature of Medicine Clinical Terms.

^e^FCI: Faculty Clinical Informatics.

### Addressing Digital Gaps

The provision of health services in the United Kingdom needs to substantially change to meet the needs of an aging population, with a key focus on more integrated care across health and care settings to local needs [33]. This requires better collaboration between different professionals working across care sectors who have the right information to inform decision-making, with digital developments recognized as key to the transformation [33,34]. To enable this, participants emphasized the need for a single consolidated electronic patient record where all health and care professionals can read and write into and share information across traditional boundaries to deliver acute care, manage long-term conditions, and ensure patients receive the right care at the right time and place. For example, most community pharmacists in the United Kingdom do not have access to an up-to-date and comprehensive list of a patient’s medications or medical records, which hinders their ability to support individuals. However, pilot projects such as the Somerset shared care record case study (SIDeR) are underway with some promising feedback, although there is a need for further evaluation to fully understand the benefits and any unintended consequences or challenges [35]. There is a strong relationship between the maturity of digital health and comprehensive evaluation methods; therefore, this should be included as part of future locally shared care strategies to support learning and facilitate the development and adoption of systems [36]. In addition, as Cresswell et al [37] noted, there are several socio-organizational dimensions of change that must be considered to support a digitally enabled shared care agenda. They summarize key areas, such as structural and organizational complexity; variations in data management and expectations; poorly defined current shared care pathways; and issues associated with reluctance to data sharing, managing “data overload,” and configuring systems appropriately. In terms of technological dimensions, existing infrastructures and legacy systems may hinder data sharing across new technological junctions, while it may also be difficult to connect incompatible data structures and overcome supplier resistance to make distinct systems interoperable [37]. In their report, they advocated the need to map potential architectural components and designs for shared care solutions with careful consideration of their potential benefits and limitations [37].

In our study, participants highlighted that the need to undertake the process of developing shared records *with* end users to ensure the design and functionality is “fit for purpose.” Specifically, participants raised the question of developing tailored solutions, that is, a bespoke community pharmacy view of a patient’s health record, to enhance the usability and utility of such systems. Research has shown how the design of EHRs can influence behavior and prescribing safety [38,39]; therefore, further work is needed to explore how to create usable health records for a range of different end users working across sectors.

Participants also expressed specific ways in which data could be used to better support the transfer of care, for instance, sending digital referrals or requests for follow-up directly between existing clinical systems, further demonstrating the need for continued engagement and collaboration with end users involved in the delivery of frontline services to optimize and enhance systems over time [40].

To fully harness the benefits of a comprehensive and consolidated shared patient record, there is a need to address digital gaps within organizations across the health and social care sector. The effectiveness and utility of the tool depends on the data within. First, organizations must prioritize the use of established and approved digital information standards related to medicines and clinical information within NHS digital systems. Notably, all NHS care providers who are involved in prescribing, dispensing, or administering medicines must transfer medication information using the newest UK version of fast health care interoperability resources, use approved dose syntax to transfer the amount of medication per dose as a simple coded quantity, and use SNOMED and dm+d codes for allergy or intolerance information by March 31, 2023 [41]. However, barriers to the adoption of such standards have been identified, including a lack of cohesive national-scale digital health system; funding and support for standards; knowledge and infrastructure related, such as the impact on preexisting workflows; and lack of use of a consistent patient ID [42,43]. Therefore, local ICSs must explore how they can address such challenges and support the implementation of the standards across the region. This underscores the importance of effective clinical leadership and understanding the personal factors that influence health IT uptake [44,45]. The adoption of digitized health records, electronic prescriptions, and medication administration across primary, secondary, and social care is vital. As is embedding digital technologies across social care, particularly as estimates suggest that less than half of social care providers have any form of digital care records in England [46]. Furthermore, research has shown that a large proportion of medication errors occur in care homes [47]; therefore, tools to support the digitization of the sector to support medicines optimization and enhance the safety, quality, and efficiency are urgently needed. Our findings echo those of a recently published report by the Royal Pharmaceutical Society Scotland, *Pharmacy 2030: a professional vision document*, which outlines the changes to and enhanced roles of pharmacy professionals and key enablers, including data to inform decision-making, harnessing digital technology, developing the workforce, and increasing emphasis on multidisciplinary work [34]. A further report and policy review from the Royal Pharmaceutical Society and The King’s Fund that will inform the development of vision for pharmacy was published at the end of 2022.

To support the delivery of enhanced medicines optimization activities across care settings, there will be an increased emphasis on pharmacists and pharmacy staff undertaking clinical roles in all sectors. Community pharmacies, for example, will have a far greater role in providing enhanced clinical services and supporting the holistic prevention of ill health in a community [48,49]. The supply of medicines will be facilitated by accuracy checking technology, such as dispensing robots, or possibly through hub-and-spoke model dispensing [50]. To support this, the participants in this study highlighted the need for changes in how services are funded through the community pharmacy contractual framework. Although the demand for community pharmacies has risen since the outbreak of the Covid-19 pandemic, staff shortages are a growing concern; 1 survey reported that 91% (estimated from responses from 418 representatives of 5000 pharmacy premises) of pharmacies have experienced staff shortages [51]. There are also huge concerns around staffing shortages more widely across the NHS, which has an impact on patient care, while awaiting the results and recommendations of the NHS long-term workforce strategy [52]. This is important because changes resulting from the implementation of new digital tools, particularly systems with poor usability, can contribute to clinician burnout and consequently reduce job satisfaction, quality and safety of care, and costs [53]. Any digital transformation relating to how medicines optimization is delivered should, therefore, be mindful of the working environment and additional stressors present, and improving the working life of health and social care providers should be a goal and actively monitored [54]. In addition, IT systems management is needed to closely monitor the number of digital systems used across an ICS footprint and rationalize how services are delivered to reduce unnecessary duplication and streamline services. This may be supported by forums and established groups with a key objective of proactively collating and communicating examples of good practices between different clinical and IT stakeholders.

### Limitations

We acknowledge some limitations of this study; semistructured interviews were conducted via web-based videocall platforms, which enabled participants across a large geographical area to participate; however, we did encounter some technical issues, which may have impacted the flow and nature of discussions. We only included 2 GPs in this study; however, 5 primary care and clinical commissioning group pharmacists who provided in-depth detail about the experiences of medicines optimization of health care professionals working within GP practices were recruited, and data were collected until thematic saturation was reached. Further work may specifically explore the challenges experienced within different sectors and enhanced by using observational data collection approaches. Furthermore, the interviewer was professionally known to a small number of participants before the study, and her experience as a practicing pharmacist and academic researcher enabled her to build rapport and relationships with the participants. Throughout data collection and analysis, notes were recorded on any personal reactions or reflections to support consideration of the intersubjective reflexivity between herself and the participants [55]. This was used during the analysis stage to provide context and allowed the researcher to honestly and critically reflect on their role in interpreting the data. It also prompted further exploration of ideas and themes during the data collection stage [55]. We collected data from a range of participants across different sectors; however, this was limited to the NENC regions and so may not be representative of other parts of the United Kingdom. Finally, although the analysis and results were discussed with the research team and member checking was performed with the participants, only 1 researcher coded the data collection transcripts, which could have further decreased the rigor and replicability of our work.

### Conclusions

The findings from this qualitative study of 23 clinical and IT stakeholders identified major complexity in terms of the number of different systems used throughout the NENC region and identified several important challenges in the transfer of care issues, focusing on having access to incomplete patient records. We also highlighted important barriers related to the use of digital tools, such as multiple systems and workflow, interoperability, digital gaps, IT systems management, and change management. Finally, participants discussed their future hopes and opportunities for the provision of medicines optimization services in the future, and there was a clear need for a patient-centered consolidated integrated health record for use by health and care professionals across different sectors, which would be fundamental to delivering effective and safe patient care. Further specific priorities were around understanding the vision for pharmacy services and supporting it with appropriate funding arrangements and strategic planning of the workforce, adoption of digital information standards, and development of minimal system requirements and frameworks. In addition, IT system management to reduce unnecessary repetition and, importantly, meaningful and continued collaboration with stakeholders and system suppliers to optimize systems and share good practices across care sectors were important key enablers.

## Appendix

Multimedia Appendix 1 Simplified overview of digital tools used across North East and North Cumbria to support medicines optimization activities.

## Abbreviations

dm+d: dictionary of medicines and devices
EHR: electronic health record
GNCR: Great North Care Record
GP: general practitioner
ICS: integrated care system
NENC: North East and North Cumbria
NHS: National Health Service
SNOMED: Systematized Nomenclature of Medicine
