# Supplementary material for: Barriers and Opportunities for the Use of Digital Tools in Medicines Optimization Across the Interfaces of Care: Stakeholder Interviews in the United Kingdom
Source: JMIR Med Inform. 2023 Mar 10;11:e42458. doi: 10.2196/42458 (PMC10039399; doi:10.2196/42458)
Supplement: Multimedia Appendix 1 [file medinform_v11i1e42458_app1.docx]

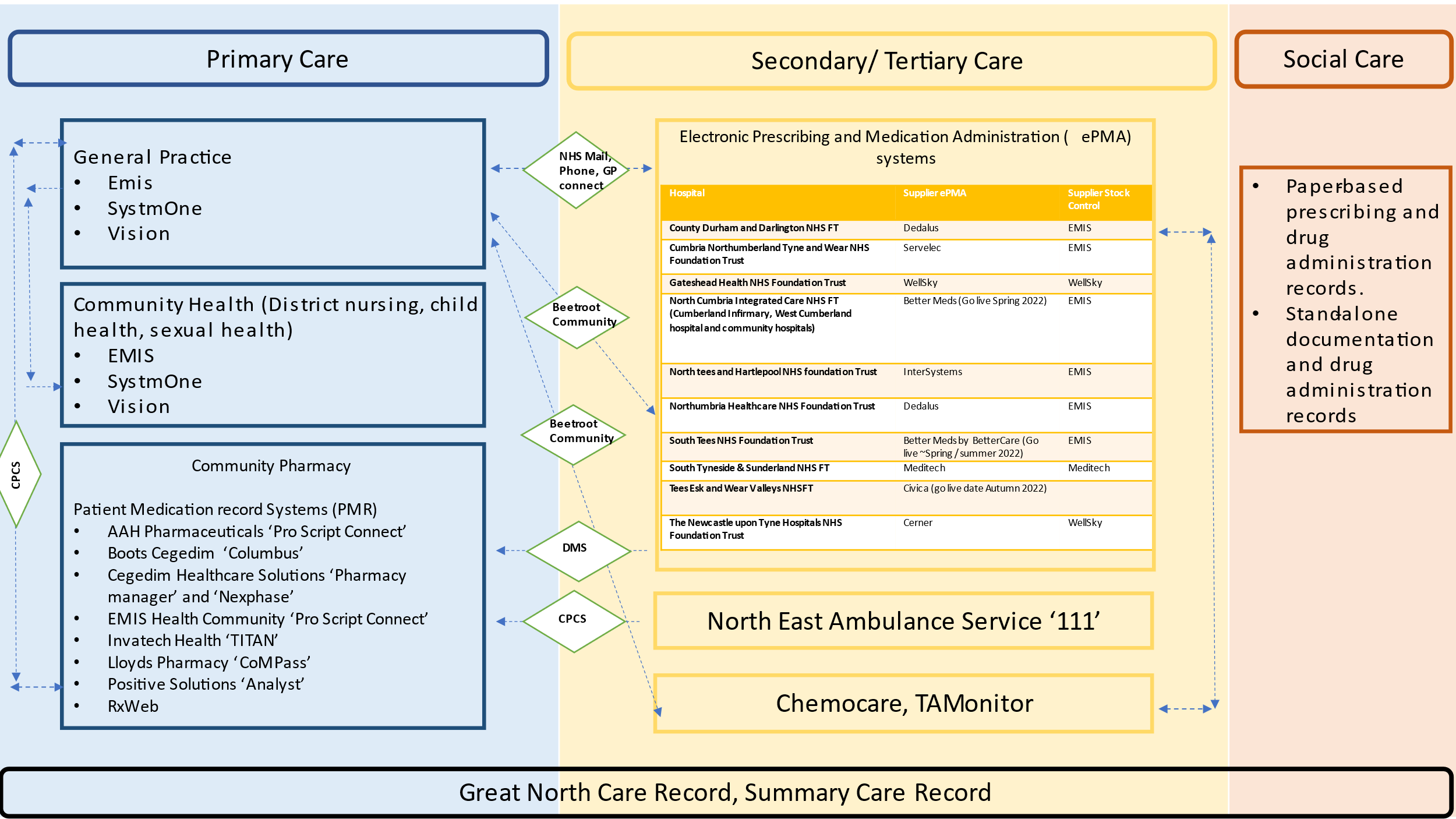


Figure 1: Simplified overview of digital tools used across NENC to support Medicines Optimisation activities

- Arrows describe the flow of medication-related information and diamonds describe the systems or services used to facilitate the flow of medication-related data.
- BeetrootCOMMUNITY is a digital tool used to support monitoring of patients prescribed Immune Modifying Drugs as part of a Shared Care Agreement (SCA): The platform can be used to share SCA between providers, monitor blood tests and prescriptions and provide automated clinical alerts[30]. A SCA is a formal local agreement that enables General Practitioners (GPs) to accept responsibility for the safe prescribing and monitoring of specialist medicines.
- TAMonitor is a digital platform used in secondary care to support monitoring of patients prescribed IMDs and provide automated alerts for clinical signs of deterioration, potential adverse events[31].
- Chemocare is a digital platform for prescribing, preparing and administering chemotherapy[32].
- Discharge Medicines Service (DMS)
  - Information and referrals sent via
    - PharmOutcomes
    - NHS Mail
- NHS Community Pharmacist Consultation Service (CPCS)
  - Information and referrals sent via
    - PharmOutcomes
    - Cegedim
    - HxConsult (Positive Solutions)
    - Sonar Informatics
